# Causal effects of gut microbiome on HIV infection: a two-sample mendelian randomization analysis

**DOI:** 10.1186/s12879-024-09176-5

**Published:** 2024-03-04

**Authors:** Kangjie Li, Cong Zhang, Jielian Deng, Haijiao Zeng, Yuan Zhang, Guichuan Lai, Xiaoni Zhong, Biao Xie

**Affiliations:** College of Public Health, Chongqing Medical University, Chongqing, China

**Keywords:** Gut microbiome, HIV infection, Mendelian randomization, Causality

## Abstract

**Background:**

The causal association between gut microbiome and HIV infection remains to be elucidated. We conducted a two-sample mendelian randomization analysis to estimate the causality between gut microbiome and HIV infection.

**Methods:**

Publicly released genome-wide association studies summary data were collected to perform the mendelian analysis. The GWAS summary data of gut microbiome was retrieved from the MiBioGen consortium, which contains 18 340 samples from 24 cohorts. GWAS summary data of HIV infection was collected from the R5 release of FinnGen consortium, including 357 HIV infected cases and 218 435 controls. The SNPs were selected as instrumental variables according to our selection rules. And SNPs with a F-statistics less than ten were regarded as weak instrumental variables and excluded. Mendelian randomization analysis was conducted by five methods, including inverse variance weighted (IVW), MR-Egger, weighted median, weighted mode, and simple mode. The Cochran’s Q test and MR-Egger intercept test were performed to identify heterogeneity and pleiotropy. Leave-one-out analysis were used to test the sensitivity of the results.

**Results:**

Fifteen gut microbiota taxa showed causal effects on HIV infection according to the MR methods. Four taxa were observed to increase the risk of HIV infection, including *Ruminococcaceae* (OR: 2.468[1.043, 5.842], P: 0.039), *Ruminococcaceae* *UCG005* (OR: 2.051[1.048, 4.011], P: 0.036), *Subdoligranulum* (OR: 3.957[1.762, 8.887], *P* < 0.001) and *Victivallis* (OR: 1.605[1.012, 2.547], *P*=0.044). *Erysipelotrichaceae* was protective factor of HIV infection (OR: 0.278[0.106, 0.731], *P* < 0.001) and *Methanobrevibacter* was also found to be associated with reduced risk of HIV infection (OR: 0.509[0.265, 0.980], *P*=0.043). Horizontal pleiotropy was found for *Fusicatenibacter* (*P*<0.05) according to the MR-Egger regression intercept analysis. No heterogeneity was detected.

**Conclusion:**

Our results demonstrate significant causal effects of gut microbiome on HIV infection. These findings facilitate future studies to develop better strategies for HIV prophylaxis through gut microbiome regulation. Further explorations are also warranted to dissect the mechanism of how gut microbiome affects HIV susceptibility.

**Supplementary Information:**

The online version contains supplementary material available at 10.1186/s12879-024-09176-5.

## Background

Human immunodeficiency virus (HIV) was firstly isolated from lymph node in 1983, which caused acquired immune deficiency syndrome [1]. At present, HIV infection has been globally pandemic, with near 39 million people living with HIV [2]. In China, the prevalence of new HIV infection was increasing [3], especially among men who have sex with men (MSM) [4]. Mechanically, HIV interacts with receptor CD4 and coreceptor CCR5 to entry T cells, allowing the virus to replicate [5]. Exposure to the semen or mucosal surface were the most common route of HIV transmission. However, as reported, the probability of HIV transmission was only 0.01%-0.4% per sexual contact [6], suggesting that HIV infection also depends on the interaction between the virus and host. Host immune activation might facilitate HIV transmission and infection. Research showed that higher levels of inflammatory factors in male penile, including IL-6 and IL-10, contributed to HIV virus shedding [7]. Besides, a nested case-control study indicated that the rate of HIV seroconversion was significantly higher in women with higher genital inflammatory cytokines [8]. In MSM, HIV mainly transmitted through anal intercourse, thus the immune barrier of rectal mucosa plays important role in the early process of HIV infection.

Gut microbiome takes part in constituting gut immune barrier and could regulate functions of small intestinal innate lymphoid cells [9]. Recent studies found that HIV infection was associated with gut microbiome dysbiosis [10–12], with decreased α-diversity, enrichment of genus Prevotella and depletion of Bacteroides in HIV patients. Furthermore, in a prospective study gut microbiome dysbiosis was demonstrated to contribute to increased HIV susceptibility in MSM [13]. The relative abundance of family *Succinivibrionaceae*, *Erysipelotrichaceae* and *Coriobacteriaceae* were significantly higher while *Bacteroidaceae* and *Rikenellaceae* were significantly depleted in HIV sero-converters. The gut microbiome dysbiosis in HIV patients were associated with immunologic response to antiretroviral therapy [14], activation of T cells [15] and increased inflammatory factors [16]. However, the association between HIV infection and gut microbiome remains controversial. After stratifying sexual preference, HIV status was reported to have subtle effects on gut microbiome [17, 18]. Meanwhile it’s also unclear whether gut microbiome dysbiosis was induced by HIV infection or prompting to the virus infection [19]. The causal relationship between gut microbiome and HIV infection was remained to be elucidated.

Mendelian randomization (MR) is an analytic approach that utilizes genetic variants as instrumental variations to assess the causality between exposure and outcome [20]. With the help of genome-wide association studies (GWAS), MR analysis is widely used to explain the causal effects of an interested exposure on outcomes, which overcomes the potential influence of confounding factors in observational studies [21]. To our knowledge, no studies have documented the causal effects of gut microbiome on HIV infection before. Hence, this study aimed to explore the causal associations between gut microbiome and HIV infection through a two-sample mendelian randomization analysis.

## Methods

### GWAS summary data source

The GWAS summary data of gut microbiome was retrieved from the MiBioGen consortium. MiBioGen is a large-scale genome-wide meta-analysis to study the association between gut microbiome and human genetic variants, which contains 18 340 samples from 24 cohorts from the USA, Canada, Israel, South Korea, Germany, Denmark, the Netherlands, Belgium, Sweden, Finland and the UK [22]. The microbiome data were generated by 16S rRNA sequencing using an Illumina sequencing platform. Multiple hyper-variable regions of the 16S rRNA gene was sequenced, including V1-V2, V3-V4 and V4 region. After adjustment for age, sex, technical covariates and genetic principal components, 211 gut microbiome taxa had GWAS summary statistics, including 9 phyla, 16 classes, 20 orders, 35 families and 131 genera. Fifteen unknown bacteria were excluded, and 196 gut microbiotas were enrolled for the final analysis. The GWAS summary data for HIV infection were collected from the 5^th^ release of the FinnGen consortium in May 2021, composed of 357 HIV infected cases and 218 435 controls [23]. We selected the gut microbiome as the exposure and the HIV infection as the outcome.

### Instrumental variables selection

To indicate causal association between gut microbiome and HIV infection, we selected SNPs that were associated with gut microbiome at the level of *P* < 1× 10 ^-5^ as instrument variables (IVs). This value was identified as the optimal threshold in many gut microbiota-related MR research to increase the amount of genetic variance explained by the genetic predictors [24].The linkage disequilibrium (LD) across SNPs were calculated in a 10,000-kb window and the LD threshold for R^2^ was 0.001. In the condition of no SNPs shared between gut microbiome and HIV infection, proxy SNPs with *r*^2^ > 0.8 were selected to replace the original SNPs. Then F-statistics was calculated for each SNP with the following formula: F = β^2^/Se^2^, whereas β and Se are the coefficient and stand error of exposure respectively. SNPs with F-statistics < 10 were regarded as weak IVs and were discarded in the following analysis. MR pleiotropy residual sum and outlier (MR‐PRESSO) tests was also conducted to remove outlier SNPs.

### Mendelian randomization analysis

Five methods were used to perform the MR analysis, including inverse variance weighted (IVW), weighted mode, MR-egger, weighted median, and simple mode. IVW uses a meta-analysis approach combined with the Wald estimates for each SNP to obtain an overall estimate, which assumes that all SNPs are valid instruments [25]. The weighted mode produces a robust causal effects estimate when the horizontal pleiotropy existed [26]. ME-egger could provide a valid effect on the condition that all SNPs are invalid [27]. The estimates based on weighted median are consistent even when more than 50% of the information comes from invalid instrumental variables [28]. Simple mode is an unweighted causal estimation [29]. The primary method was IVW, and only when the result of IVW analysis was significant, the causal effects of exposure on outcome presented. Other four MR methods were used as supplement. Additionally, false discovery rate (FDR) was calculated based on the Benjamini-Hochberg (BH) method to adjust the multiple tests. FDR < 0.1 were considered significant [30].

**Fig. 1:**
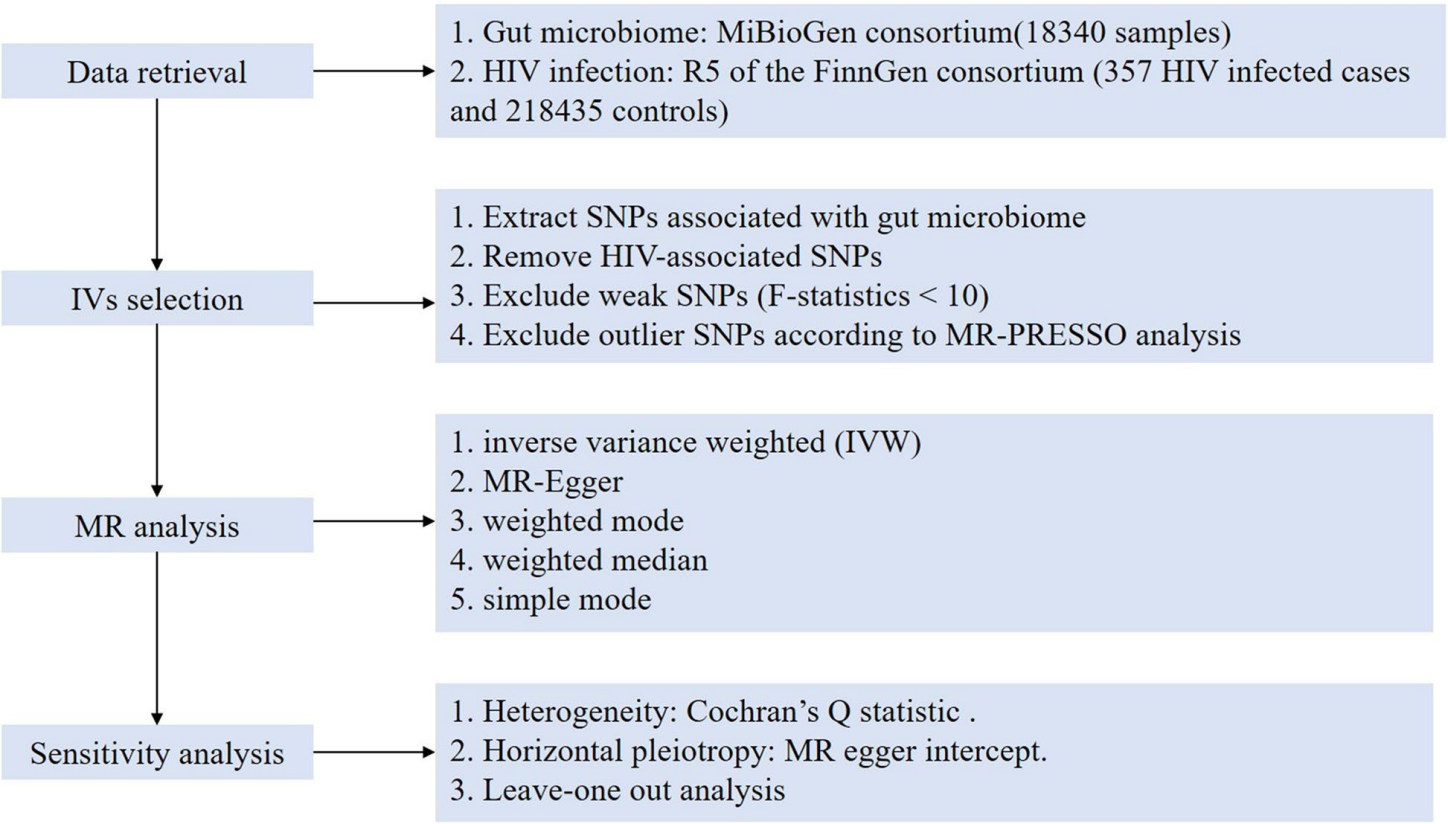
The flowchart of this mendelian randomization study

Sensitivity analysis was conducted to evaluate the stability of the results. Cochran’s Q statistic was used to test the heterogeneity. MR egger intercept was used to assess the horizontal pleiotropy. *P* value < 0.05 indicated no significant heterogeneity or horizontal pleiotropy. Besides, leave-one out analysis was conducted to identify whether the overall estimates were influenced by one single SNP. To further exclude the impact of confounding factors, such as behavior and environment, we searched the Phenoscanner database (http://www.phenoscanner.medschl.cam.ac.uk/) to explore the reported traits that were associated with our selected IVs. The study flowchart was stated in Fig. 1.

All the analysis were performed by R software (version: R 4.3.0). R packages “TwosampleMR” was used to conduct the two-sample mendelian randomization analysis. And R package “MRPRESSO” was used to identify the outlier SNPs.

## Results

### Causal effects of gut microbiome on HIV infection

At the level of *p* < 1 x 10^-5^, 2 774 SNPs were associated with gut microbiota at phylum, class, order, family, and genus levels. Fifty-three SNPs were excluded due to the F-statistic < 10. Thus, a total of 2 721 SNPs were enrolled for MR analysis. Fifteen bacteria were found to have causal effects on HIV, including 1 class, 2 orders, 3 families and 9 genera (Table 1). One genus of phylum *Actinobacteria* was negatively related to HIV infection. One class, 2 orders, 3 families and 6 genera of phylum *Firmicutes* were significantly associated with HIV infection. One genus of phylum *Lentisphaerae* and 1 genus of phylum *Methanomada* were also showed causal effects on HIV infection.

**Table1:** Causal effects of gut microbiome on HIV infection

Phylum	Taxa	Gut microbiota	Methods	NSNP	Beta	SE	OR	95%CI		*P* value	FDR	Heterogeneity		Horizontal pleiotropy		
												Cochran’s Q	*P* value	Egger intercept	SE	*P* value
Actinobacteria	genus	Eggerthella	IVW	10	-0.740	0.267	0.477	0.283	0.805	0.006	**0.089**	2.071	0.979	-0.245	0.135	0.106
Firmicutes	class	Erysipelotrichia	IVW	13	-1.280	0.493	0.278	0.106	0.731	0.009	0.232	13.602	0.256	-0.157	0.127	0.244
Firmicutes	class		Weighted median		-1.825	0.648	0.161	0.045	0.575	0.005	0.245					
Firmicutes	family	Defluviitaleaceae	IVW	11	-0.681	0.321	0.506	0.270	0.948	0.034	0.423	6.600	0.679	0.125	0.112	0.291
Firmicutes	family	Erysipelotrichaceae	IVW	13	-1.280	0.493	0.278	0.106	0.731	0.009	0.232	13.602	0.256	-0.157	0.127	0.244
Firmicutes	family		Weighted median		-1.825	0.616	0.161	0.048	0.539	0.003	0.168					
Firmicutes	family	Ruminococcaceae	IVW	9	0.903	0.440	2.468	1.043	5.842	0.040	0.452	4.266	0.749	-0.121	0.094	0.242
Firmicutes	family		Weighted median		1.214	0.597	3.365	1.045	10.836	0.042	0.736					
Firmicutes	genus	Anaerotruncus	IVW	13	-0.836	0.402	0.434	0.197	0.954	0.038	0.439	8.052	0.709	-0.068	0.080	0.415
Firmicutes	genus		Weighted median		-1.080	0.535	0.340	0.119	0.970	0.044	0.744					
Firmicutes	genus	Clostridium	IVW	7	-0.857	0.431	0.424	0.182	0.988	0.047	0.492	5.442	0.364	-0.023	0.115	0.850
Firmicutes	genus	Coprococcus2	IVW	8	-0.976	0.441	0.377	0.159	0.894	0.027	0.391	5.093	0.532	-0.141	0.249	0.591
Firmicutes	genus	Fusicatenibacter	IVW	18	-0.374	0.385	0.688	0.323	1.465	0.332	0.873	14.826	0.537	0.201	0.090	0.040
Firmicutes	genus		MR Egger		-3.254	1.338	0.039	0.003	0.531	0.027	1.000					
Firmicutes	genus	Ruminococcaceae UCG005	IVW	14	0.718	0.342	2.051	1.048	4.011	0.036	0.432	11.441	0.492	-0.021	0.078	0.797
Firmicutes	genus	Subdoligranulum	IVW	11	1.375	0.413	3.957	1.762	8.887	0.001	**0.085**	8.564	0.478	-0.043	0.078	0.592
Firmicutes	genus		Weighted median		1.455	0.580	4.286	1.376	13.354	0.012	0.445					
Firmicutes	order	Bacillales	IVW	9	-0.494	0.213	0.610	0.402	0.927	0.021	0.352	5.687	0.577	0.026	0.138	0.855
Firmicutes	order	Erysipelotrichales	IVW	13	-1.280	0.493	0.278	0.106	0.731	0.009	0.232	13.602	0.256	-0.157	0.127	0.244
Firmicutes	order		Weighted median		-1.825	0.607	0.161	0.049	0.530	0.003	0.168					
Lentisphaerae	genus	Victivallis	IVW	10	0.473	0.235	1.605	1.012	2.547	0.044	1.000	9.228	0.323	0.255	0.231	0.302
Methanomada	genus	Methanobrevibacter	IVW	6	-0.675	0.334	0.509	0.265	0.980	0.043	1.000	6.052	0.195	-0.028	0.200	0.895

At the class level, 13 SNPs were selected for genetically predicting *Erysipelotrichia*. Based on IVW analysis and weighted median analysis, *Erysipelotrichia* was protective factor of HIV infection (IVW: OR: 0.278[0.106, 0.731], *P* < 0.01; weighted median: OR: 0.161[0.045, 0.575], *P*<0.01). Order *Bacillales* and *Erysipelotrichales*, both belonged to *Firmicutes*, were significantly associated with HIV. The results of IVW analysis indicated that both *Bacillales* and *Erysipelotrichales* were protective factors of HIV (*P*<0.05). Three families of *Firmicutes*, *Defluviitaleaceae*, *Erysipelotrichaceae* and *Ruminococcaceae* had causal effects on HIV infection. *Defluviitaleaceae* and *Erysipelotrichaceae* were significantly associated with decreased risk of HIV infection, while *Ruminococcaceae* was identified as risk factor of HIV infection in IVW and weighted median analysis (IVW: OR:2.468[1.043,5.842], *P*=0.04; Weighted median: OR:3.365[1.045, 10.836], *P*=0.04.). At the genus level, *Eggerthella*, *Anaerofilum*, *Anaerotruncus*, *Clostridium*, *Coprococcus2*, *Fusicatenibacter* and *Methanobrevibacter* were protective factors. *Hungatella*, *Ruminococcaceae UCG005*, *Subdoligranulum* and *Victivallis* were risk factors of HIV infection. After adjusting for the multiple testing, genus *Eggerthella* and genus *Subdoligranulum* were found to significantly decrease the risk of HIV acquirement (FDR < 0.1). All these results indicated that gut microbiome had significant causal effects on HIV infection.

**Fig. 2:**
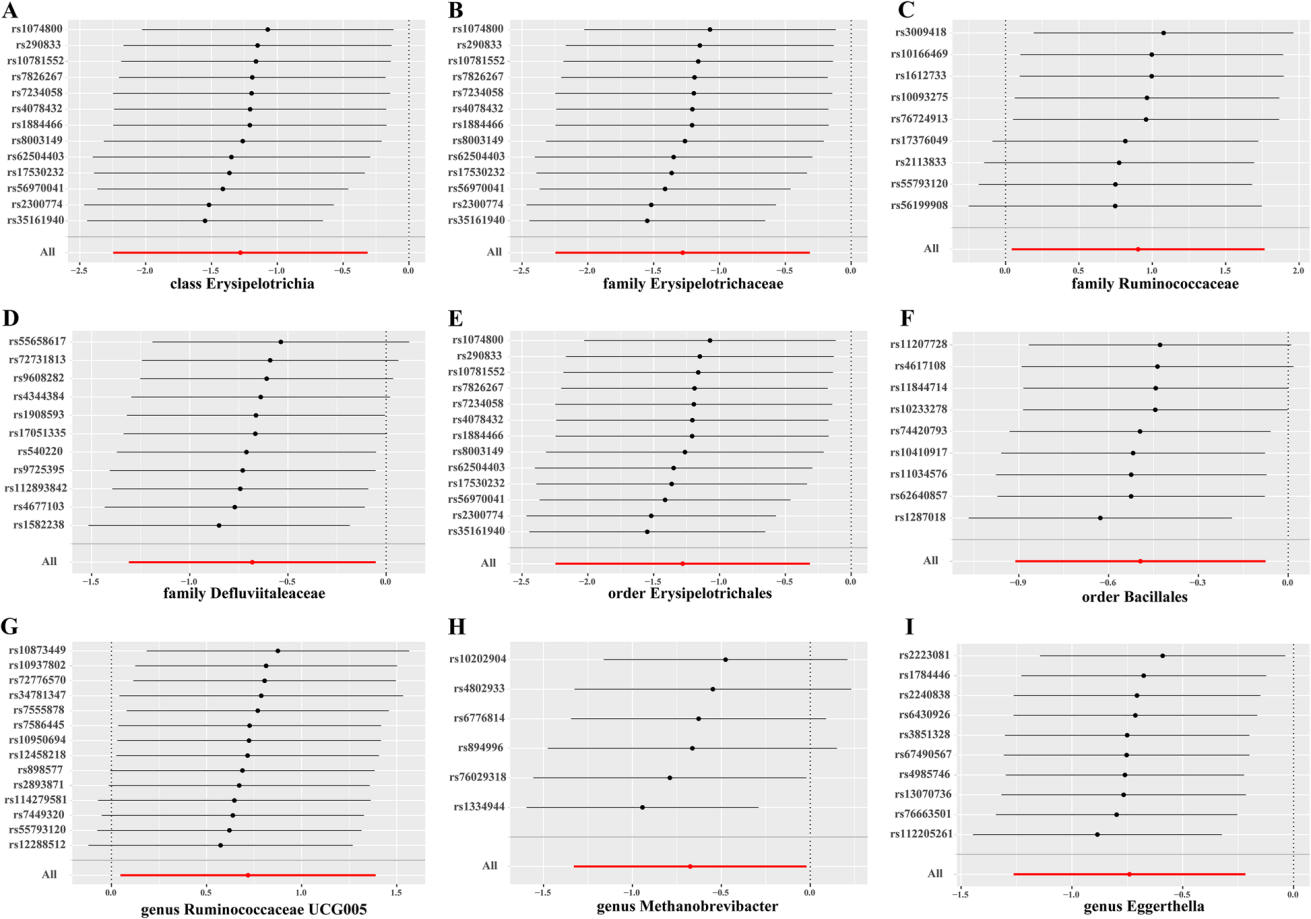
Leave-one-out analysis of the significant causal effects of gut microbiome on HIV infection. The X-axis indicates the estimated β value. In each panel, the red line stands for the overall estimates, and each blank line indicates the overall estimate after excluding the left SNP. A: class Erysipelotrichia, B: family Erysipelotrichaceae, C: family Ruminococcaceae, D: family Defluviitaleaceae, E: order Erysipelotrichales, F: order Bacillales, G: genus Ruminococcaceae UCG005, H: genus Methanobrevibacter, I: genus Eggerthella

Among all these fifteen gut microbiotas, the results of Cochran’s Q test indicated no significant heterogeneity existed. However, horizontal pleiotropy was found for genus *Fusicatenibacter* (*P*<0.05) according to the MR-Egger regression intercept analysis. Leave-one out sensitivity analysis suggested that the causal association between gut microbiome and HIV infection were not driven by one single SNP (Fig. 2, Figure S1). Besides, we searched for the Phenoscanner database to explore whether potential confounding factors existed. The results (Table S1) showed that our selected IVs were associated with a few physical indicators, such as impedance of arms, body mass index, arm fat mass, weight, height and so on. These factors were known as influencing factors of gut microbiome composition, but were not reported to affect HIV infection, which demonstrated that the causal association between gut microbiome and HIV was robust.

## Discussion

The present study was the first MR analysis to evaluate the causal relationships between gut microbiome and HIV infection. We demonstrated causal effects of 15 gut microbiotas on HIV infection. In our study, we found that family *Ruminococcaceae*, and genus *Hungatella*, *Ruminococcaceae UCG005*, *Subdoligranulum* and *Victivallis* were significantly associated with HIV infection, which might facilitate HIV infection. Genus *Clostridium*, *Coprococcus2* and *Fusicatenibacter* were identified as protective factors against HIV infection. Typically, most of our reported gut microbiotas were belonged to *Firmicutes* phylum. Importantly our results stated causality between HIV infection and family *Erysipelotrichaceae* and genus *Methanobrevibacter*, which was consistent with one prior study [13], further demonstrating that gut microbiome dysbiosis had major impact on HIV incidence. After adjusting for the multiple testing, genus *Eggerthella* and genus *Subdoligranulum* were found to significantly decrease the risk of HIV acquirement, indicating robust causal effects of gut microbiome on HIV infection.

In the field of HIV infection, gut microbiome has aroused researchers’ interests. Researchers found that the composition of gut microbiome significantly changed in HIV-infected patients [18]. To explain the mechanism how gut microbiome affect HIV infection, researches have focused on exploring the association between gut microbiome and immune. In MSM, the high-risk population of HIV infection, gut microbiome can influence the CCR5 expression and influx of HIV targeted cells to colon through modulation of integrin and chemokines expression on T cells [31]. Using gnotobiotic mice model, researchers found that gut microbiome from high-risk MSM activated T cells, with higher frequency of CD69^+^ and CD103^+^ T cells [14]. In vitro assays, gut microbiome from HIV-infected patients could induce significantly higher levels of activated monocytes and T cells, which were mediated by TNF-α and TLR (toll like receptor) [16]. Further study revealed that *Holdemanella biformis* was the main signature that was responsible for elevated frequency of CCR5^+^ CD4^+^ T cells in the vitro stimulation assay of Lamina Propria Mononuclear Cells with fecal bacterial communities [32]. All these evidences demonstrated gut microbiome had the potential of increasing HIV transmission through activating immune reaction.

In the present study, family *Erysipelotrichaceae* decreased risk of HIV infection (OR: 0.28, *P* < 0.05), which means that higher the relative abundance of *Erysipelotrichaceae* protects patients against HIV invasion. As reported members of this bacterial were highly immunogenic, for example *Erysipelotrichi* was reported to correlate with TNF in chronic HIV infection patients [33]. Besides, *Erysipelotrichaceae* has been found to associate with inflammatory bowel diseases (IBD) and colon cancers [34]. Actually, *Erysipelotrichaceae* is a kind of butyrate-producing bacteria [35]. Butyrate is the primary energy source of colon epithelial cells and plays important role on ameliorating mucosal inflammation and oxidative status, reinforcing the epithelial defense barrier to maintain intestinal health [36]. Moreover, butyrate is a well-known histone deacetylase inhibitor and could regulate gene expression through modulating chromatin structure, indicating its potential therapeutic implication for clinical use [37]. Studies documented that oral butyrate supplementation in patients with IBD activated epithelial peroxisome proliferator-activated receptor-γ (PPAR-γ) signaling and promoted generation of Treg cells in colonic lamina propria [38]. Animal experiment also demonstrated that oral butyrate downregulated inflammation in mice implanted with polyether-polyurethane sponge, presented with decreased neutrophil infiltration and reduced levels of TNF-α, IL-10 and TGF-β1 [39]. All these evidences shed light on the hypothesis that *Erysipelotrichaceae* decreased the risk of HIV infection through enhancing the production of butyrate which inhibited excessive activation of immune system. Meanwhile, *Methanobrevibacter* was also showed protective effects on HIV infection in our analysis. *Methanobrevibacter* belongs to methanogens and plays important physiological role in human health by producing methane from hydrogen [40]. Study indicated that *Methanobrevibacter smithii* colonized in the human gastric mucosa just early after birth [41]. *Methanobrevibacter* was documented to be associated with irritable bowel diseases and possibly reversed the susceptibility of irritable bowel diseases [42, 43]. However, further research is needed on the specific relationship between *Methanobrevibacter* and host health.

Moreover, we also found *Subdoligranulum* and *Victivallis* were positively related to HIV infection. Consistent with previous studies, *Subdoligranulum* and *Victivallis* were enriched in HIV infected patients, which further supporting our results [11, 44]. Interestingly, contrary to *Erysipelotrichaceae*, butyrate-producing bacteria *Ruminococcaceae* was demonstrated to increase the risk of HIV infection in our study. This conclusion seems to be contradictory, but in fact it implies the complex interaction between gut microbiome and suggests that the effects of gut microbiome on HIV infection are not simply caused by single microbiota. This also indicates that the structure of gut microbiome is in dynamic balance, and only when the balance is broken will it affects health of the host.

What is worth to note is that genus *Prevotella* was not statistically correlated with HIV infection in this MR analysis. However, *Prevotella* was the most frequently reported HIV associated genus and might promote to virus infection. For example, researchers found that the abundance of *Prevotella* was higher in women with high-risk HPV infection and associated with NF-KB signaling, suggesting its role in promoting virus infection by altering immune regulators [45]. Besides, the abundance of *Prevotella* was associated with immune response, which could drive Th17-mediated mucosal inflammation and stimulate epithelial cells to produce inflammatory factors, such as IL-8 and IL-6 [46]. The impact of genus *Prevotella* on HIV infection remains to be further elucidated.

To be honest, there are limitations in this study. Firstly, the results of reverse MR analysis exploring causal effects of HIV infection on gut microbiome was not stated here. In fact, we indeed conducted the reverse MR analysis and attempted to explore the causal effects of HIV infection on gut microbiome. However, there are no SNPs available as instrumental variables in the currently used summary GWAS data of HIV infection. On the other hand, we also tried using the latest release HIV GWAS summary data of FinnGen consortium in May 2023, but still lack of available instrumental variables. Secondly, we recommend to treat the significant results carefully. We adjusted the *P* values based on BH method to avoid false discovery and only two taxa passed the BH correction (FDR < 0.1). More studies are expected to elucidate the role of gut microbiome in HIV infection.

## Conclusions

In conclusion, this study analyzed the causal association between gut microbiome and HIV infection by a two-sample MR analysis. Our results demonstrate that several gut taxa increase the risk of HIV acquirement, such as *Ruminococcaceae*, *Subdoligranulum* and *Victivallis*. While *Erysipelotrichaceae* and *Methanobrevibacter* significantly reduce the risk of HIV infection. We hold the concept that the risk of HIV infection may not be significantly increased by one single microbiome, it is the results of complex interaction of variable gut microbiome. These findings facilitate future studies to develop better strategies for HIV prophylaxis through gut microbiome regulation. Further explorations are also warranted to dissect the mechanism of how gut microbiome affects HIV susceptibility.

### Supplementary Information

**Supplementary Material 1.** 

**Supplementary Material 2.** 

## Data Availability

The datasets analyzed during the current study are available in the Finngen database (https://www.finngen.fi/en) and MiBioGen database (https://mibiogen.gcc.rug.nl/), which are publicly available.

## Abbreviations

GWAS: Genome-wide association studies
IVW: Inverse variance weighted
HIV: Human immunodeficiency virus
MSM: Men who have sex with men
MR: Mendelian randomization
IVs: Instrument variables
LD: Linkage disequilibrium
MR‐PRESSO: MR pleiotropy residual sum and outlier
FDR: False discovery rate
