# Supplementary material for: Causal effects of gut microbiome on HIV infection: a two-sample mendelian randomization analysis
Source: BMC Infect Dis. 2024 Mar 4;24:280. doi: 10.1186/s12879-024-09176-5 (PMC10913272; doi:10.1186/s12879-024-09176-5)
Supplement: Supplementary file 1 — Supplementary Material 1. [file 12879_2024_9176_MOESM1_ESM.pdf]

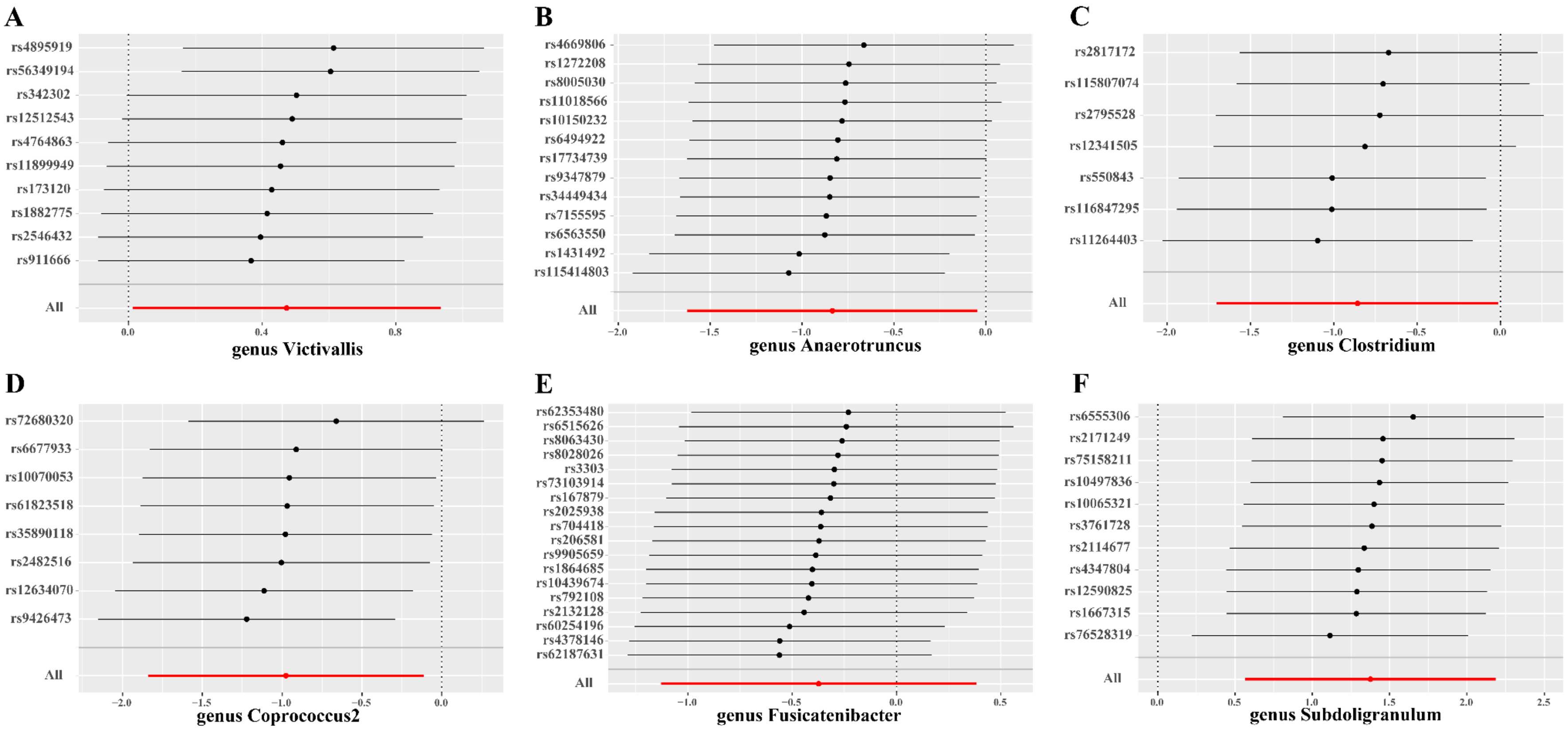

Figure S1. Leave-one-out analysis of the significant causal effects of gut microbiome on HIV infection. The X-axis indicates the estimated β value. In each panel, the red line stands for the overall estimates, and each blank line indicates the overall estimate after excluding the left SNP. A: genus Victivallis, B: genus Anaerotruncus, C: genus Clostridium, D: genus Coprococcus2, E: genus Fusicatenibacter, F: genus Subdoligranulum.
